# Bilberry-Derived Anthocyanins Modulate Cytokine Expression in the Intestine of Patients with Ulcerative Colitis

**DOI:** 10.1371/journal.pone.0154817

**Published:** 2016-05-06

**Authors:** Sofia Roth, Marianne R. Spalinger, Claudia Gottier, Luc Biedermann, Jonas Zeitz, Silvia Lang, Achim Weber, Gerhard Rogler, Michael Scharl

**Affiliations:** 1 Division of Gastroenterology and Hepatology, University Hospital Zurich, Zurich, Switzerland; 2 Institute for Surgical Pathology, University Hospital Zürich, Zurich, Switzerland; 3 Zurich Center for Integrative Human Physiology, University of Zurich, Zurich, Switzerland; Laikon Hospital, GREECE

## Abstract

**Background/Aims:**

We previously demonstrated that anthocyanin-rich bilberry extract (ARBE) inhibits IFN-γ-induced signalling and downstream effects in human monocytic cells and ameliorates disease activity in ulcerative colitis (UC) patients. Here, we studied the molecular mechanisms of ARBE-mediated effects *in vitro* and by analysing colonic tissue and serum samples of UC patients treated with an oral anthocyanin-rich bilberry preparation during an open label clinical trial.

**Methods:**

Colon specimens obtained during an open pilot study using ARBE for the treatment of mild-to-moderate UC were analyzed by immunohistochemistry. Cytokine levels in patients’ serum were quantified by ELISA. Cell culture experiments were performed using THP-1 monocytic cells.

**Results:**

ARBE treatment inhibited the expression of IFN-γ-receptor 2 in human THP-1 monocytic cells. Colon biopsies of UC patients who responded to the 6-week long ARBE treatment revealed reduced amounts of the pro-inflammatory cytokines IFN-γ and TNF-α. Levels of phosphorylated (activated) p65-NF-κB were reduced in these patients. Further, patients with successful ARBE treatment featured enhanced levels of Th17-cell specific cytokine IL-22 and immunoregulatory cytokine IL-10 as well as reduced serum levels of TNF-α and MCP-1, but enhanced levels of IL-17A, in contrast to patients that did not reach remission after ARBE treatment.

**Conclusions:**

Our data suggest a molecular mechanism underlying the anti-inflammatory effects of ARBE treatment in UC patients by modulating T-cell cytokine signalling and inhibiting IFN-γ signal transduction. These data are of particular interest, since ARBE is a promising therapeutic approach for the treatment of IBD.

## Introduction

Phenols are plant-derived molecules with anti-inflammatory, anti-oxidant, anti-carcinogenic, anti-adipogenic and neuroprotective properties [1,2]. Chemically, they consist of one or more (*poly*phenols) aromatic ring(s) with at least one hydroxyl group attached. Based on their chemical structure, they are classified into two groups, flavonoids and non-flavonoids. Anthocyanidins form a crucial sub-class of dietary flavonoids and are widespread in fruits and flowers where they account for the blue, purple and red colours. In these plant-derived forms they are commonly conjugated to sugars or organic acids and consequently named anthocyanins [1,2]. Our regular diet contains a variety of natural phenols. Especially berries, red wines, leafy and root vegetables and certain whole grain cereals comprise relative high amounts of anthocyanins. Due to their health-promoting and protective characteristics substantial interest in phenols has emerged lately [3]. Multiple epidemiological studies demonstrated that polyphenol-rich foods prevented diseases like coronary heart disease, certain types of cancer and inflammatory diseases [4,5,6]. The benefits of polyphenols were originally attributed to their anti-oxidant properties. Nevertheless, additional mechanisms such as direct interference with receptor-regulated signalling pathways and gene expression have been postulated [7,8,9].

Ulcerative colitis (UC), a sub-form of inflammatory bowel disease (IBD), is characterized by a chronic and relapsing immune-mediated inflammation of the colon initiated by a dys-regulated immune response to commensal intestinal microbiota and environmental factors in the genetically susceptible host [10,11,12]. Prevalence and incidence of IBD have increased in western countries as well as in less developed countries where lower incidence rates were reported previously [13]. The fact that numerous IBD patients are not satisfactorily treated with the established treatment options or suffer from therapy-related side effects aggravates the burden of IBD [14,15]. Thus, there is a need for more effective, well-tolerated and safe therapeutic options.

Bilberries (Vaccinium myrtillus L.)–closely related to blueberries (Vaccinium corymbosum)–have one of the highest natural anthocyanin content [16]. In a previous study [17] we have confirmed the finding that anthocyanin-rich extracts reduce inflammatory gene expression *in vitro* [16,18,19]. Moreover, it has been demonstrated that bilberry ingestion and subsequently anthocyanin intake attenuates the severity of experimental colitis and diminishes pro-inflammatory cytokine serum levels in animal models [20,21,22,23]. Based on these findings, our group conducted an open label pilot study in patients with mild to moderate UC (approved by the local ethics committee (EK-1733), trial not registered) [24]. In addition to their standard medication, patients were treated with an anthocyanin-rich bilberry preparation. After 6 weeks, endoscopic and histologic disease activity and fecal calprotectin levels were significantly reduced in the study participants, therefore anthocyanins represent a potential therapeutic option in IBD. In this study, we aimed to further investigate the molecular processes underlying the protective properties of anthocyanins. On one hand, we conducted in vitro experiments with human monocytic THP-1 cells. On the other hand, we further analysed colon biopsies and serum samples from UC patients who had participated in the above discussed open label pilot study by Biedermann et al. [24] focussing on the expression of T-cell derived cytokines.

## Materials and Methods

### Reagents and Antibodies

All reagents were of analytical grade and obtained commercially. Monoclonal rabbit anti-human phospho-STAT1 (Tyr^701^; D4A7), polyclonal rabbit anti-human STAT1 were obtained from Cell Signaling Technologies (Danvers, MA, United States).

Human recombinant IFN-γ was obtained from Sigma (Sigma-Aldrich, St. Louis, MO, United States). The anthocyanin-rich bilberry extract (ARBE) was manufactured by Kaden Biochemicals, Symrise GmbH & Co (Holzminden, Germany) and was allocated as a powder (25% anthocyanin content). This powder was dissolved in ddH_2_O to establish a stock suspension of 10 mg ARBE/ml. Due to sedimentation, the stock suspension was homogenized by strong agitation during 30 minutes prior to each usage. A detailed analysis of the ARBE powder can be found in S1 File.

### Patient Samples

Intestinal tissue specimens were taken during the open label bilberry ingestion pilot study by Biedermann et al. [24]. 13 patients with current mild to moderate UC underwent a first sigmoidoscopy with biopsy taking 7 days prior to and 11 patients underwent a second sigmoidoscopy at the last day of the six-week bilberry intake period as described in the aforementioned study [24]. Patient’s serum was taken at the study visits 7 days prior to the beginning of the bilberry treatment (baseline = week 1) and from there on in a 2-week interval (i.e. week 3, week 5 and at the last day of the bilberry intake at week 7). Serum was frozen at -80°C and used for ELISA experiments.

### THP-1 Cell Culture, Vector Transduction, Phosphatase Inhibition and Stimulation Protocols

Human monocytic THP-1 cells (DSMZ no. ACC 16, DSMZ, Braunschweig, Germany) were cultured in RPMI 1640 medium (Life technologies, Gibco, Carlsbad, CA, United States) supplemented with 10% fetal calf serum (FCS) at an approximate density of 0.5 to 1 x 10^6^ cells/ml. Cells were maintained in a 5% CO_2_ and 95% humidified incubator at 37°C. For experiments without siRNA transfection, cells were seeded in 1 ml of FCS-free RPMI 1640 medium + 1% Penicillin/Streptomycin per well at a density of 1 to 1.5 x 10^6^ cells/ml at least 6 hours prior to treatment.

PTPN2 siRNA transfection was performed using 100 pmol of a gene specific siRNA oligonucleotide (Life technologies) and the Amaxa nuclefector system (Lonza, Walkersville, MD, United States) according to the manufacturer’s instructions. After transfection, THP-1 cells were cultured in a 12-well plate for 48 hours prior to treatment.

Pre-treatment with ARBE solution (composed of ARBE stock suspension and FCS-free RPMI 1640 medium + 1% Penicillin/Streptomycin) with a final concentration of 10 μg/ml was conducted 20 min before stimulation. Then, IFN-γ was applied in a previously validated concentration of 100 ng/ml ([25] and S1 Fig) for either 30 min (Western blot experiments) or 24 h (qPCR experiments).

Inhibition of cellular phosphatases was induced by Na_3_VO_4_ (Sigma-Aldrich, S6508) according to manufacturer’s recommendations and Gordon J.A. [26]. Na_3_VO_4_ in a final concentration of 0.5 mM was added to the THP-1 cells simultaneously with ARBE pre-treatment.

### Preparation of Whole Cell Lysates

THP-1 cells were washed twice with phosphate buffered saline (PBS) and lysed in M-PER Mammalian protein extraction reagent (Pierce Biotechnology, Rockford, IL, United States) containing protease inhibitors (Roche, Basel, Switzerland) for 45 min. After centrifugation (10 min at 13,000 g), cell lysate supernatants were assayed for protein content using a NanoDrop spectrophotometer (NanoDrop ND1000; Pierce Biotechnology).

### Western Blotting

Each lysate was mixed with loading buffer (NuPAGE® 4x LDS Sample Buffer (Life technologies), 500mM dithiothreitol and boiled for 5 min at 95°C. Separation of the proteins was performed with SDS-polyacrylamide gel electrophoresis (SDS-PAGE). Subsequently, the proteins were transferred onto nitrocellulose membranes (Millipore, Billerica, MA, United States).

Membranes were blocked during 1 h with blocking solution (3% milk powder (C. Roth GmbH+Co. KG, Karlsruhe, Germany) and 1% bovine serum albumin (BSA) (GE Healthcare, PAA Laboratories GmbH, Pasching, Austria) in washing buffer (Tris buffered saline containing 1% Tween 20). Primary antibody was diluted in blocking solution (1:1000 for all experiments). Membranes were incubated in primary antibody solution overnight at 4°C and then washed with washing buffer for 30 min. Horseradish peroxidase (HRP)-labelled secondary anti-mouse- or anti-rabbit-IgG-antibody (1:5000; sc-2005, sc-2004; Santa Cruz Biotechnologies, Inc., Dallas, TX, United States) in blocking solution was added for 1 h and membranes were washed again for 30 min. Immunoreactive proteins were detected using an enhanced chemiluminescence detection kit (Thermo Scientific, Rockford, IL, United States), and exposure on X-ray films (GE Healthcare, Little Chalfont, UK). Films were scanned and intensity of the protein bands determined using NIH ImageJ software. Densitometry values from proteins were normalized to the corresponding values from non-treated controls. For STAT1, densitometry values from phosphorylated proteins were additionally normalized to the values from the corresponding total proteins.

### RNA Isolation and Complementary DNA Synthesis

THP-1 cells were washed with ice-cold phosphate buffered saline (PBS) and disrupted in RLT buffer (Qiagen, Venlo, The Netherlands) and 1 M dithiothreitol solution. Total RNA was isolated using RNeasy Mini Kit (Qiagen) according to manufacturer’s instructions. RNA concentration was measured by absorbance at 260 nm (NanoDrop ND1000). Complementary DNA (cDNA) was synthesised using a High-Capacity cDNA Reverse Transcription Kit (Applied Biosystems, Foster City, CA, United States) following the manufacturer’s recommendations.

### Real-time Polymerase Chain Reaction (PCR)

Real-time PCR was performed using FAST qPCR Master Mix for Taqman Assays (life technologies) on a Fast 7900HT Real-Time PCR system using SDS Software (life technologies). Measurements were performed in triplicate, human β-actin was used as endogenous control, and results were analyzed by ΔΔCT method. The real-time PCR contained 45 cycles consisting of a denaturing (95°C, 1 sec) and an annealing/extending (60°C, 20 sec) step. Gene expression assays were all obtained from life technologies.

### Immunohistochemistry

Immunohistochemistral procedures were performed using colon biopsies of eleven study participants with UC. The biopsies were taken in the context of the bilberry study by Biedermann et al. [24]. Additionally, colon biopsies from healthy controls (n = 4), from UC patients with active colitis (n = 1–2) and from UC patients with colitis in remission under conventional therapy (n = 1–2) were investigated. A peroxide-based method on the formalin-fixed and paraffin-embedded tissue specimens was applied. First, the tissue was deparaffinised with HistoClear® (Chemie Brunschwig AG, Basel, Switzerland) and rehydrated in descending concentrations of ethanol (100%, 96%, 70%), double-distilled water and PBS pH 7.2. In order to retrieve the target proteins and to avoid cross-linking reactions the biopsies were boiled in a citrate buffer solution (10mM, pH 6.0, DAKO, Glostrup, Denmark) in a 98°C hot water bath for 30 min. The specimens were cooled down at room temperature and buffer traces were removed by washing the tissue twice in PBS. For inactivation of the numerous endogenous peroxidases present in intestinal tissue the specimens were incubated in a 0.9% H_2_O_2_ solution for 15 min at room temperature. After another two washing steps in PBS the specimens were blocked with 1–3% BSA in PBS for 1 h in a humidified chamber at room temperature. Subsequently, the tissue was incubated with polyclonal rabbit anti-human IFN-γ (H-145) IgG (dilution 1:300; concentration 200 μg/ml; sc-8308, Santa Cruz Biotechnology), polyclonal Rabbit anti-human IFN-γ R1 (dilution 1:100; concentration 0.5 mg/ml; HPA029213, Sigma-Aldrich), polyclonal Rabbit anti-human IFN-γ R2 (dilution 1:20; concentration 0.5 mg/ml; HPA001535, Sigma-Aldrich) polyclonal rabbit anti-human Stat1 (dilution 1:400; #9172S, Cell Signaling), monoclonal mouse anti-human TNF-α (52B83) IgG_1_ (dilution 1:100; concentration 100μg/ml; sc-52746, Santa Cruz Biotechnology), monoclonal rabbit anti-human phospho-NF-κB p65 (Ser^536^; 93H1) IgG (dilution 1:100; #3033S, Cell Signaling), monoclonal rat anti-human IL10 (JES3-12G8) IgG_2a_ (dilution 1:100, concentration 0.5 mg/ml; Thermo Fisher, Pierce Biotechnology), monoclonal mouse anti-human IL17A (aa1-75, 4K5F6) IgG_2b,k_ (application 7 μg/ml, concentration 0.5 mg/ml; LS-B8323; LifeSpan BioSciences, Inc., Seattle, WA, United States), or polyclonal rabbit anti-human IL22 (dilution 1:500, concentration 1.0 mg/ml; NB-100-737, Novus Biologicals, Littleton, CO, United States), overnight at 4°C in a humidified chamber. After two rinsing steps in PBS the tissue was incubated with corresponding secondary antibodies (ImmPRESS^TM^ HRP anti-mouse (MP-7402)/anti-rabbit (MP-7401) Ig (peroxidase) polymer detection kit from Vector laboratories, Inc., Burlingame, CA, United States; goat anti-rat IgG-HRP, sc-2006, Santa Cruz Biotechnology) for 1 h at room temperature in a wet chamber. After another two washing steps in PBS we used the 3,3′-diaminobenzidine (DAB) method according to the manufacturer’s instructions (ImmPACT DAB peroxidase (HRP) Substrate, SK-4105, Vector laboratories) for visualization and subsequently rinsed the tissue again in PBS. For counterstaining Mayer’s hemalaun solution (Cantonal Pharmacy, Zurich, Switzerland) was applied and the tissue was then dehydrated in ascending concentrations of ethanol and Histoclear®. The results of the staining were analyzed using an AxioCam MRc5 (Zeiss, Jena, Germany) on a Zeiss Axiophot microscope (Zeiss) with Axio Vision Release 4.7.2 software (Zeiss). For quantification, we performed blinded scoring and rated with no (-), few (+), moderate (++) and intense (+++) staining.

### Enzyme-linked Immunosorbent Assay (ELISA)

The participant’s serum was stored at -80°C. ELISA kits detecting human TNF-α (PK-EL-63707), human MCP-1 (PK-EL-64006), human IL-10 (PK-EL-62006), human IL-13 (PK-EL-62306) and human IL-17A (PK-EL-62716) were obtained from Promokine (Heidelberg, Germany), human IFN-γ ELISA kit (SEH00380A) was purchased at Qiagen. IL-22 was detected with an ELISA set (DY-782-05) from R&D Systems, Inc. (Minneapolis, MN, United States). Assays were carried out following the manufacturer’s instructions using a sample volume of 50 and 100 μl, respectively. Absorbance at stated wavelengths was detected on a BioTek-Synergy II Multi-Mode Reader using Gen5.1.11 Software. All measurements were performed in duplicate.

### Statistical Analysis

Data are presented as means ± SEM for a series of n experiments. Data are expressed as relative values of the respective control. Statistical analysis was performed by analysis of variance (ANOVA) followed by Student-Newman-Keuls post hoc test. p values < 0.05 were considered significant.

## Results

### ARBE prevents the IFN-γ-induced expression of IFN-γ receptor 2 in vitro

**Fig 1:**
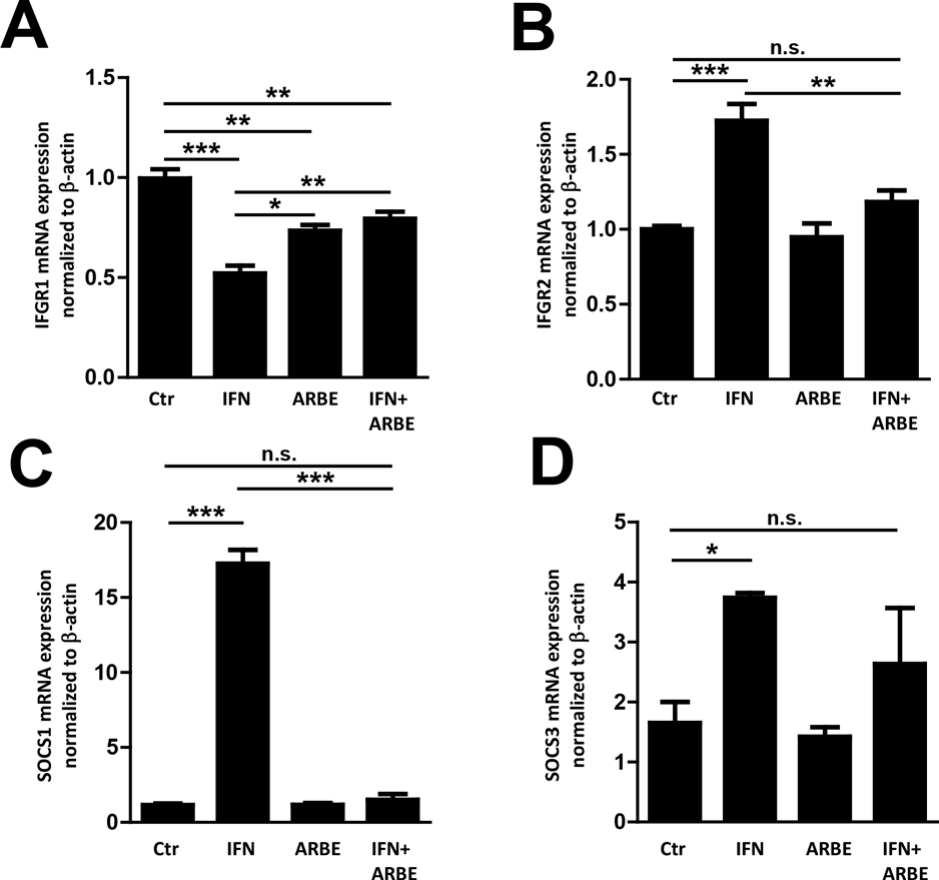
ARBE prevents the induction of IFN-γ receptor 2 (IFGR2) expression by IFN-γ. Administration of 100 ng/ml IFN-γ to THP-1 cells provoked a significant reduction of IFN-γ receptor 1 (IFGR1) that is crucial for binding of IFN-γ to its receptor **(A)**. On the other hand, IFGR2 that mediates intracellular signal transduction was significantly induced upon the stimulation with IFN-γ **(B)**. Interestingly, these effects were suppressed when cells were co-treated with 10 μg/ml ARBE (IFN+ARBE). IFGR2 expression in untreated control cells (Ctr) was comparable to co-treated cells (IFN+ARBE) **(B)**. The expression of SOCS1 and 3 was induced by IFN-γ stimulation while co-treatment with ARBE led to reduced expression of SOCS1 and 3 **(C+D)**. Significant results are marked as follows: * = p<0.05; ** = p<0.01; *** = p<0.001; n.s. = not significant. Error bars depict SEM. Ctr = control, IFN = IFN-γ.

To study the molecular mechanisms of ARBE-induced reduction of IFN-γ signalling–mainly STAT1 phosphorylation–as well as cytokine expression and secretion [17], human THP-1 cells were treated with IFN-γ (100 ng/ml) and/or ARBE (10 μg/ml). As expected, IFN-γ treatment reduced the expression of IFN-γ receptor 1 (IFN-γ R1), but enhanced levels of IFN-γ R2. Interestingly, co-treatment with ARBE diminished the effect of IFN-γ treatment on the mRNA expression of IFN-γ R1 (albeit its expression was still below the level in untreated cells) and completely prevented IFN-γ-mediated induction of IFN-γ R2 (Fig 1A and 1B). (Raw data for all experiments are shown in S2 File.) This is of particular interest, since IFN-γ R1 is important for binding of IFN-γ to its receptor and IFN-γ R2 mediates signal transduction via the intracellular JAK-STAT pathway activation after IFN-γ binding. The IFN-γ induced JAK-STAT pathway mainly involves STAT1 phosphorylation, resulting in target gene expression [27,28].

We next assessed whether ARBE co-treatment might also impact STAT1 phosphorylation by affecting the expression of SOCS1 and 3. As anticipated, IFN-γ treatment induced mRNA levels of SOCS1 as well as of SOCS3. However, the IFN-γ-mediated induction of SOCS1 mRNA was completely abrogated in ARBE co-treated cells. A similar trend was also observed for SOCS3 expression (Fig 1C and 1D).

**Fig 2:**
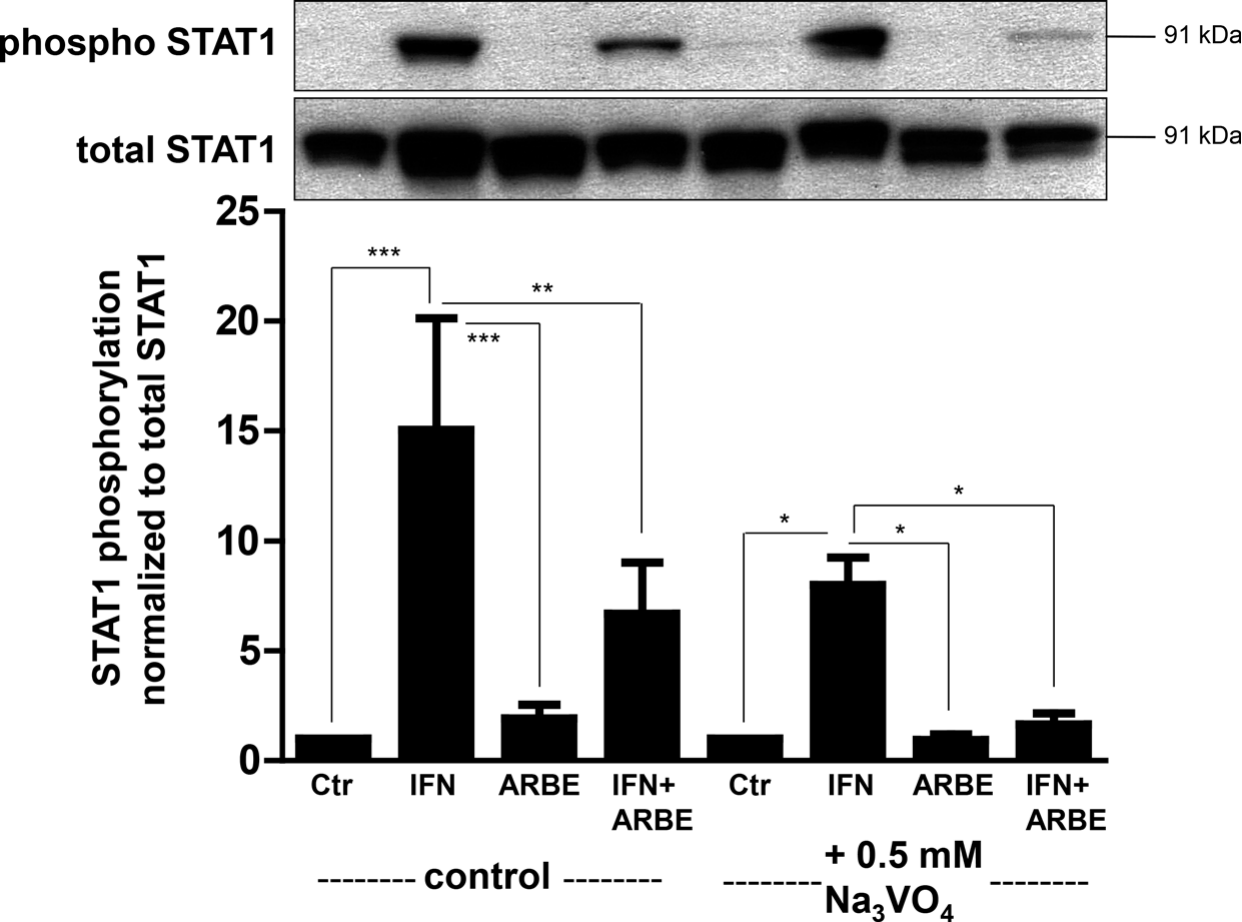
Inhibition of protein tyrosine phosphatases (PTPs) in THP-1 cells does not prevent ARBE-mediated reduction of IFN-γ-induced STAT1 phosphorylation. Application of 0.5mM Na_3_VO_4_ (PTP inhibitor) did not abrogate ARBE-induced reduction of IFN-γ-mediated STAT1 phosphorylation suggesting that ARBE does not activate PTPs. Asterisks denote significant differences (* = p<0.05; ** = p<0.01; *** = p<0.001). N = 3. Error bars depict SEM. Ctr = control, IFN = IFN-γ.

A further mechanism of STAT1 regulation is via protein tyrosine phosphatase (PTP) activity. To assess whether ARBE might inhibit phosphorylation of STAT1 via activation of PTPs, we used the PTP inhibitor Na_3_VO_4_. However, PTP inhibition had no impact on the ARBE-mediated inhibition of IFN-γ-induced STAT1 phosphorylation (Fig 2, S2 Fig). Confirmatory, siRNA-induced knock-down of protein tyrosine phosphatase non-receptor type 2 (PTPN2), the nuclear phosphatase of STAT1, did not affect the ARBE-mediated inhibition of the IFN-γ-induced effects on STAT1 phosphorylation either (S3 Fig), suggesting that PTPs do not play a role for the ARBE-mediated inhibitory effects on IFN-γ-induced STAT1 phosphorylation.

### IFN-γ and IFN-γ R2 expression in colon tissue decreases during ARBE treatment in vivo

To further elucidate the anti-inflammatory properties of ARBE in the context of human disease, we performed immunohistochemical analysis of several biological markers in human colonic biopsy specimens from 11 UC patients who had completed the bilberry ingestion study of Biedermann et al. [24]. For each patient one biopsy before and one after 6 weeks of daily bilberry intake was analysed. Additionally, several cytokine concentrations were measured in the participants’ serum by the ELISA method.

**Fig 3:**
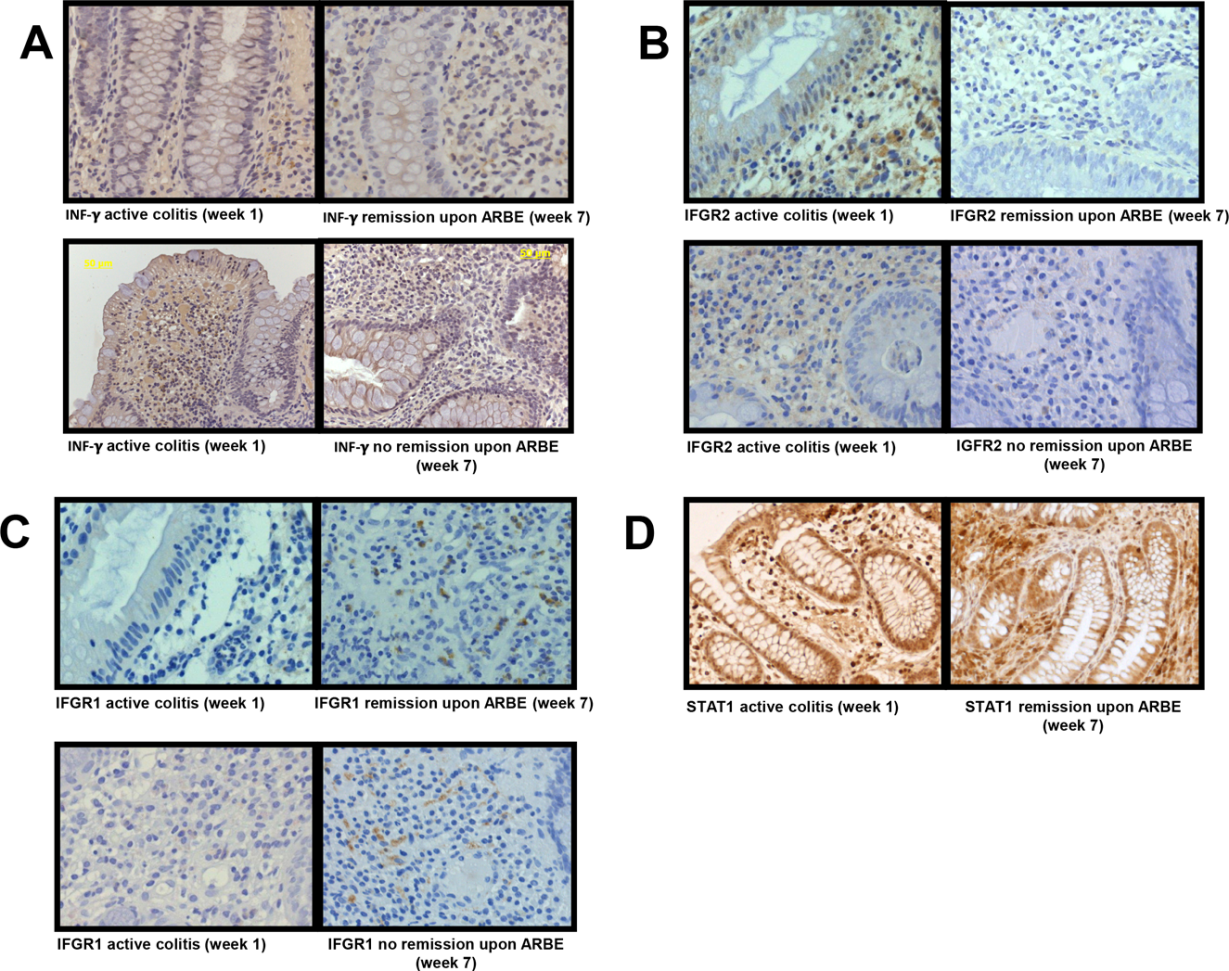
Reduced IFGR2 expression in colon tissue after 6 weeks of bilberry ingestion. Representative pictures from colon biopsies taken before (week 1) and after six weeks of daily bilberry treatment (week 7) from patients reaching remission or not. (**A**) shows specific staining for IFN-γ where a decline of IFN-γ expression was registered for patients in remission only. A marked decline in IFGR2 expression was provoked by ARBE ingestion not dependent on the remission status (**B**). A moderate enhancement in IFGR1 expression was detectable, both in patients that reached remission upon ARBE treatment as well as those that did not (**C**). Expression of total STAT1 (especially in the L. propria mucosae) decreases in patients reaching remission after 6 weeks of daily bilberry ingestion (**D**).

Since our previous findings revealed an impressive reduction of IFN-γ-related cytokine expression/secretion upon ARBE treatment *in vitro* [17], we focused on the investigation of the IFN-γ-JAK/STAT-pathway in this study. Analysis of IFN-γ staining revealed an–especially submucosal–diminution of IFN-γ expression at the end of the bilberry intake period in patients in remission, whereas no alteration was detectable in patients without clinical remission (Fig 3A, S4A Fig, S1 Table). In healthy control specimens, IFN-γ expression was generally low or not evident (S5A Fig, S1 Table). On the other hand, IFN-γ serum concentrations–that were generally very low–were not affected by ARBE treatment (S6A Fig). In addition, there was no correlation between the IFN-γ serum concentrations and the clinical activity index (CAI).

At begin of the study, IFN-γ R2 expression was readily detectable in submucosal and intestinal epithelial cells. Consistent with our cell culture data, six weeks anthocyanin intake reduced IFN-γ R2 expression in the colon of the study participants to levels comparable to healthy controls. Interestingly, this reduction was observed in all patients, independent of the remission status (Fig 3B, S4B Fig, S1 Table). On the contrary, consistently high IFN-γ R2 expression was observed in the active colitis and colitis in remission control biopsies (S5B Fig, S1 Table).

On the other hand, levels of IFN-γ R1 were low in intestinal biopsies taken before start of the ARBE regimen (and in active colitis control biopsies). However, anthocyanin ingestion resulted in submucosal accumulation of IFN-γ R1 positive cells–comparable to healthy controls–, disregarding the remission status (Fig 3C, S4C Fig, S1 Table). Though, control specimens from patients with colitis in remission upon conventional therapy did not show an accumulation of IFN-γ R1 positive cells (S5C Fig, S1 Table).

We next analysed the expression of total STAT1 in the colon biopsies. Coherently with the decline of IFN-γ expression, the number of total STAT1 expressing cells decreased to healthy control levels during the study course in patients reaching remission (Fig 3D, S4D and S5D Figs, S1 Table).

### TNF-α expression in colon tissue declines depending remission status

**Fig 4:**
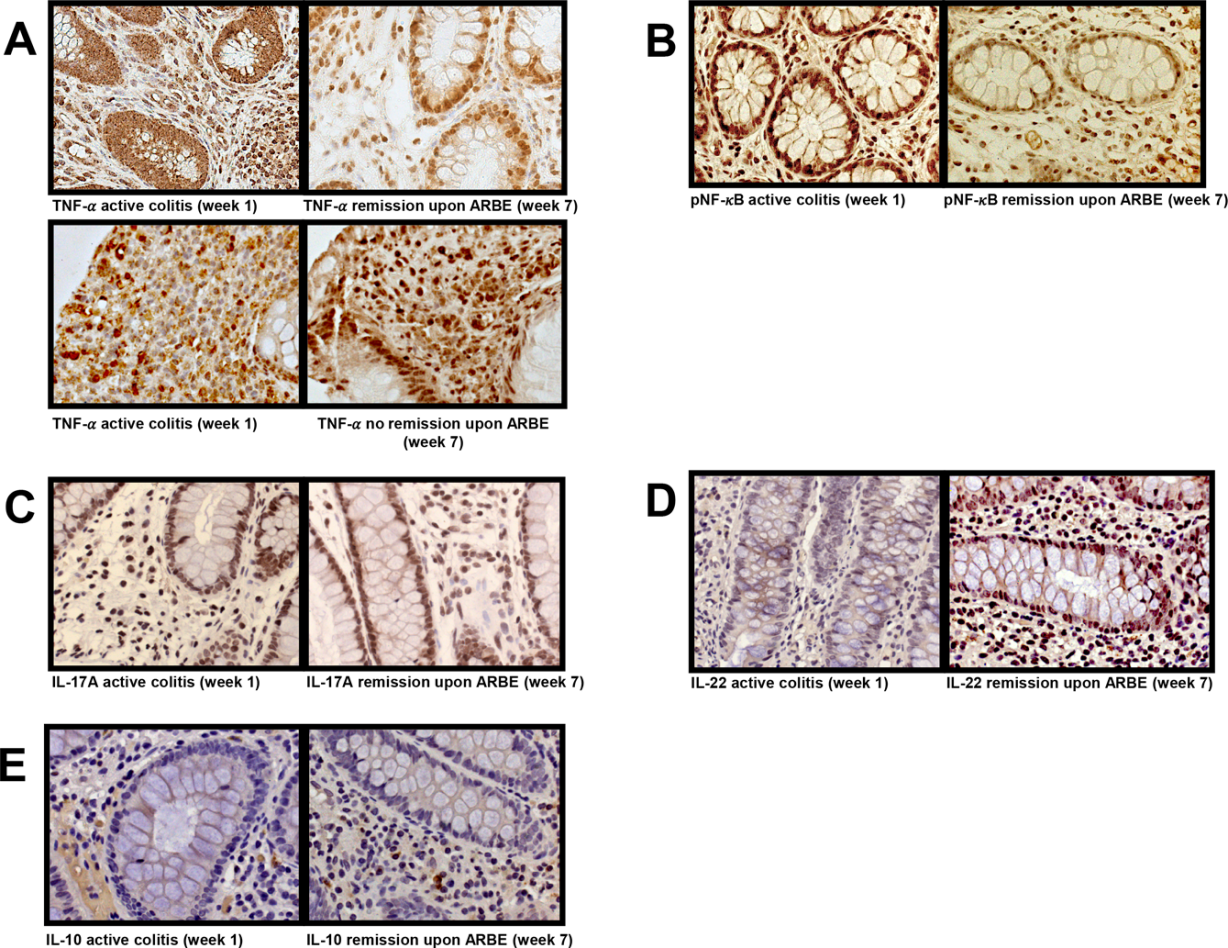
A decrease in TNF-α expression is registered in patients reaching remission only. Images illustrate representative pictures from colon biopsies taken before (week 1) and after (week 7) ARBE treatment period. TNF-α expression regressed in patients that reached remission, whereas expression levels remained stable when no remission was reached upon ARBE treatment (**A**). phospho-p65-NF-κB expression at the end of the study was reduced in some but not all patients reaching remission under ARBE treatment (**B**). Anthocyanins had no definite effect on the expression of IL-17A in colon tissue (**C**). Yet, IL-17A expression levels were constantly higher than in healthy control colon tissue. An increase in colonic IL-22 expression was detectable within the study course in patients reaching remission (**D**). IL-10 expression levels increase moderately during the study course (**E**), nearly approaching healthy control levels.

Since our previous in vitro studies demonstrated that ARBE exerts pro- and anti-inflammatory properties in response to TNF-α in human immune cells [17], we further investigated the impact of dietary anthocyanin intake on TNF-α levels in colon tissue and serum. In patients reaching remission, there was a decline in TNF-α expression to levels similar as in healthy controls after six weeks of bilberry treatment, while in patients not reaching remission, no reduction in TNF-α expression was observed (Fig 4A, S7A Fig, S1 Table). As expected, TNF-α levels in the active colitis control specimen were elevated, whereas they were lowered in a biopsy from a UC patient in remission upon conventional therapy (S8A Fig, S1 Table). This indicates that the reduction in TNF-α levels is dependent on remission status and not a direct result from ARBE treatment. A similar trend was found in serum concentrations where TNF-α decreased in patients in remission (n = 3; from 555 pg/ml down to 90 pg/ml), whereas they stayed stable when remission was not achieved (n = 2; from 3041.72 pg/ml to 2967 pg/ml) (S6B Fig).

TNF-α causes phosphorylation (activation) of the intracellular transcription factor NF-κB which is the major signal transducer of TNF-α [29]. Therefore, we next performed a phospho-p65-NF-κB staining (Fig 4B, S7B Fig, S1 Table). Reduction of phospho-p65-NF-κB positive cells was observed in some patients reaching remission, yet levels were still above healthy control levels and comparable to the levels in colitis in remission upon conventional therapy (S8B Fig, S1 Table).

### Bilberry intake reduced MCP-1 serum levels

**Fig 5:**
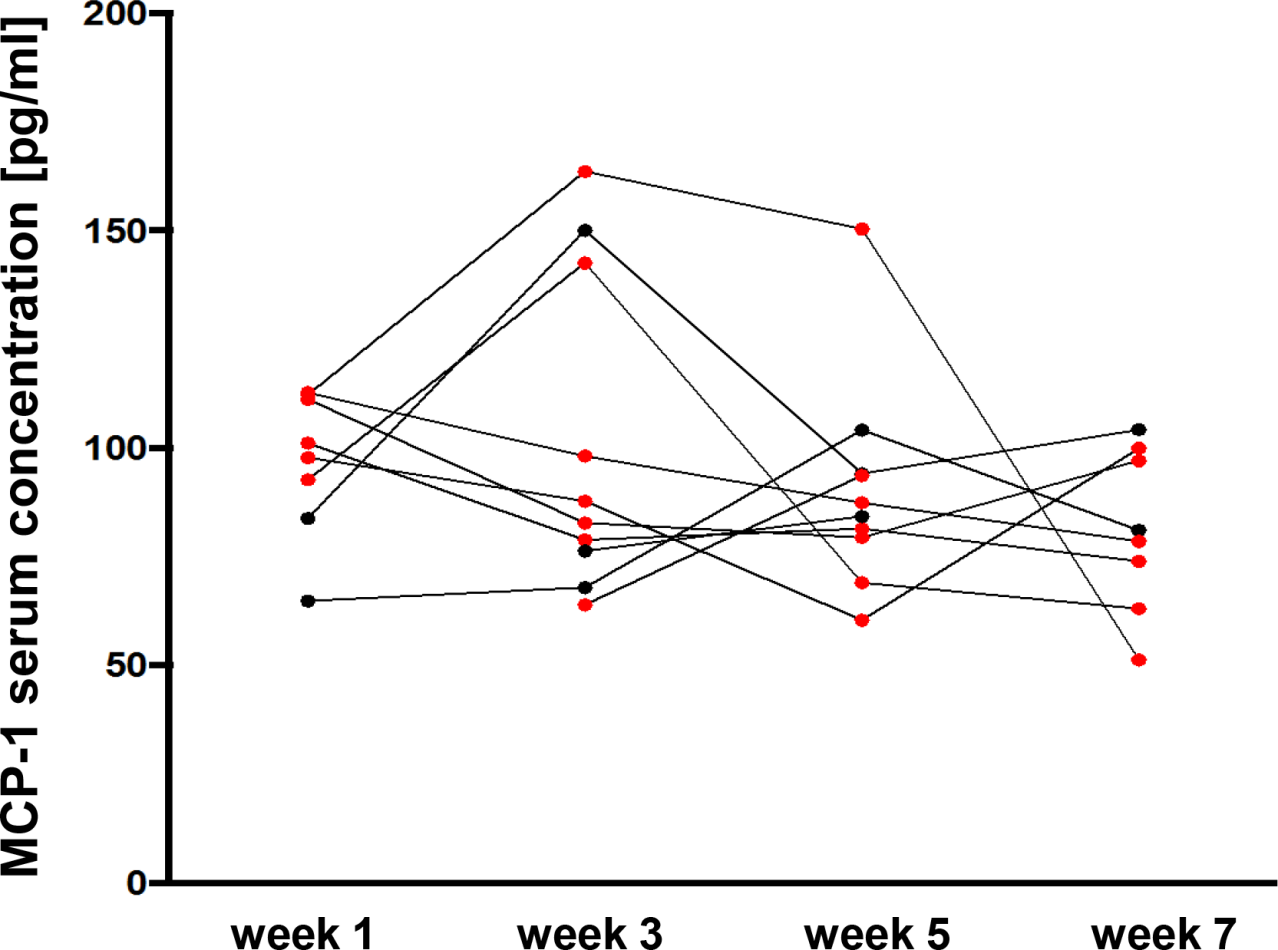
Serum concentrations of the pro-inflammatory cytokine MCP-1 declined during the study course. Analysis of MCP-1 serum levels was performed at the beginning of the bilberry intake (week 1) and subsequently in intervals of 2 weeks (week 3, week 5 and week 7). Red dots are derived from patients that reached remission (n = 7) at the end of the study whereas black dots belong to patients that did not reach remission (n = 3). Overall, a decline in MCP-1 serum levels at the end of the study was registered only in patients reaching remission.

Next, we analysed serum levels of the pro-inflammatory cytokine MCP-1 (Fig 5). Overall, patients being in remission (n = 6) featured a decline in MCP-1 serum levels at the end of the study (105 pg/ml down to 77 pg/ml), while patients not reaching remission (n = 2) at week 7 even revealed increased MCP-1 serum levels (74 pg/ml vs. 93 pg/ml). Remarkably, the two patients with infliximab treatment before study inclusion started with relative high MCP-1 levels, showed a transient rise and finally ended with the lowest MCP-1 concentrations among all the participants.

### Bilberry intake reduced Th17-specific cytokine protein expression while their secretion was not affected

We next studied the impact of the daily bilberry intake with respect to the protein expression of the typical Th17 cytokines IL-17A and IL-22 in the colon. As expected, UC patients featured elevated IL-17A expression compared to healthy control colon tissue (Fig 4C, S7C and S9A Figs, S1 Table). ARBE treatment did not result in a definite change in IL-17A expression, whilst IL-17A serum levels increased when remission was reached (n = 5; 0,53 pg/ml vs 0,8 pg/ml) or remained stable in patients without remission (n = 2) (S6C Fig).

IL-22 expression was enhanced under ARBE treatment when remission was reached. Yet, active colitis and colitis in remission upon conventional therapy also featured elevated IL-22 expression levels compared to healthy controls (Fig 4D, S7D and S9B Figs, S1 Table). Serum IL-22 levels were rather stable during the study period (S6D Fig).

### Bilberry ingestion enhanced IL-10 expression

We next addressed the impact of ARBE-enriched diet on IL-10 and IL-13. IL-10 colon expression either increased slightly or remained unaltered, yet levels remained under the healthy control levels (Fig 4E, S7E and S9C Figs, S1 Table). Although, IL-10 serum levels were generally very low, an increase of the IL-10 serum concentrations was detected in 5 of 8 patients (S6E Fig).

For IL-13, only serum concentrations were measured. Results were inconclusive since only 3 patients featured concentrations above the detection limits (S6F Fig).

## Discussion

In this investigation on the biomolecular mechanisms underlying the anti-inflammatory properties of ARBE in UC patients, we found that ARBE suppresses IFN-γ-induced expression of IFN-γ R2 in human monocytic cells (THP-1) and reduces expression of IFN-γ R2 in the intestine of UC patients. Colon biopsies of UC patients undergoing successful ARBE treatment further revealed a modified composition of T-cell-derived cytokines.

There is increasing evidence for the attenuating potential of polyphenols on IBD disease severity in humans and in experimental colitis in mice/rats [20,21,22,24]. Here, we analysed study material of the first non-controlled clinical intervention study investigating the efficacy of an oral anthocyanin-rich bilberry preparation in 11 patients with mild to moderate UC performed by Biedermann et al. [24]. This study reported significant beneficial effects on the inflammatory disease activity. The aim of our investigations was to gain insights into the cytokine expression of the colon and the serum cytokine levels during that 6-week long study period, to ultimately permit conclusions on the molecular mechanisms of the applied bilberry-derived anthocyanins.

The cytokine profile between UC and CD shows some characteristic differences. Based on the levels of T cell-derived cytokines in IBD mucosa, it has traditionally been postulated that CD is driven by an abnormal Th1 response, while UC has been thought to be a Th2-mediated disease. Accordingly, higher amounts of IFN-γ –a Th1-derived cytokine–have been reported in CD mucosa compared to UC whereas UC has been associated with elevated IL-5 and IL-13 levels possibly derived from Th2 cells. However, this traditional view was to some extent rejected when increasing evidence for the crucial role of Th17 cells emerged [30,31]. Th17 cells are involved in the inflammation of both, UC and CD, and their presence in the colon is associated with excessive expression levels of Th17 cytokines such as IL-17A, IL-17F and IL-22 [32,33].

In this study we investigated IFN-γ in context of UC–which seems contradictory at first sight–since we found a remarkable inhibition of IFN-γ-derived cytokine expression and secretion upon ARBE application in our preceding cell culture experiments [17]. Additionally, IFN-γ remains an important cytokine in UC pathogenesis and elevated IFN-γ expression correlates with disease activity [34,35,36].

IFN-γ effects are mainly elicited through the JAK/STAT pathway [28]. Here, we found that ARBE inhibits the IFN-γ-induced expression of IFN-γ R2, which is the signal transducing part of the IFN-γ receptor. Reduced levels of this molecule might well explain, how ARBE can act in an anti-inflammatory manner by inhibiting IFN-γ-induced pro-inflammatory effects. Additionally, we found that ARBE does not promote expression of SOCS family proteins that are responsible for the negative feedback mechanism of STAT activation [7].

The importance of TNF-α in the pathogenesis of both, UC and CD, is fortified by the impressive results of clinical trials targeting TNF-α [37]. Moreover, TNF-α levels correlate with disease severity [31,38,39]. In this study, we demonstrated that daily bilberry ingestion provoked a reduction in IFN-γ and TNF-α levels in the intestinal tissue in those patients who reached remission while the levels did not decrease in those participants without remission. Since reduction of IFN-γ and TNF-α was only observed in patients reaching remission after the trial period, but not in patients consuming bilberry extract without reaching remission, this effect might be the results of reduced disease activity and not a direct effect of the ARBE ingestion. This is further strengthened since patients in remission after conventional therapy show a similar reduction of IFN-γ and TNF-α. Further, the serum levels of these cytokines were not affected in a conclusive way. As ingestion of anthocyanin-rich bilberry extract resulted in reduced expression of IFN-γ R2 and STAT1 not depending on the remission status, these effects are likely to be mediated directly by ARBE treatment.

As described above, a specific subset of Th cells–Th17 cells–has been reported to play a key pathogenic role in inflammatory conditions, including IBD. Th17 cells are characterized by their ability to produce high amounts of IL-17A, IL-17F, IL-22, IL-10 and other cytokines crucial for host defence against extracellular bacteria and fungi. In IBD, the intestinal mucosa is characterized by a massive infiltration of Th17 cells and consequently reveals excessive amounts of Th17-related cytokines [33,40]. However, neutralization of IL-17A (by antibody treatment or genetic knockdown) leads to exacerbated intestinal inflammation in the dextran sulphate sodium (DSS) colitis model [41,42]. Therefore, tissue-protective properties of Th17 cytokines are assumed. In our study, we observed an increase in IL-17A and IL-22 levels in the intestinal tissue and/or serum after the bilberry intake period. This is of particular interest and well in line with results from a clinical proof-of-concept trial using the anti-IL-17A monoclonal antibody secukinumab. Here, inhibition of IL-17A by secukinumab has been demonstrated to cause fatal effects in IBD patients [43]. All in all, more and more data suggest that Th17 cytokines IL-17 and IL-22 might be rather protective for the intestine than deleterious. In our study, we have analysed the effects of ARBE treatment in UC patients only and in our knowledge no investigations on ARBE in CD patients have been conducted so far. Yet, there is increasing evidence of the involvement of Th17 cells and their cytokines in the pathogenesis of CD suggesting common molecular mechanisms in these two disease entities [44,45]. Therefore, a potential benefit of ARBE in the treatment of CD patients remains to be assessed.

The regulatory T-cell (Treg) cytokine IL-10 is a crucial immunosuppressive cytokine produced by a variety of leukocytes and non-hematopoietic cells and features a central role in the regulation of intestinal mucosal homeostasis and prevention of IBD [46,47]. However, according to the literature inflamed intestinal tissue comprises increased numbers of mononuclear IL-10 producing cells [48,49] accompanied by increased IL-10 serum levels [50]. Our findings were in good accordance with these data, since we found an increase in IL-10 expression in patients being in remission after treatment with ARBE. The induction of IL-10 might be an important additional mechanism how ARBE exert anti-inflammatory effects in vivo.

In summary, we provide the first translational human study investigating the molecular mechanisms of action of ARBE in UC patients. Our findings are based on an open label human pilot study and therefore only *in vitro* data provided controlled results. On the one hand, ARBE directly down-regulates IFN-γ-induced effects by inhibiting IFN-γ R2 expression. On the other hand, ARBE seems to alter T-cell subsets in the intestine of UC patients. The small number of participants impedes conclusive statements about the role of ARBE in UC disease control and is a major drawback of this study. Yet, our findings are of importance for further clinical use of anthocyanins and/or ARBE, since they demonstrated one–but probably not the only—molecular mechanism how ARBE selectively modifies crucial immune mechanisms in the intestine. In conclusion, our data as well as the previous clinical trial data by Biedermann et al. [24] reinforce the possible therapeutic use of ARBE in UC patients and warrant further prospective, randomized and controlled clinical studies. Given the impressive reduction of IFN-γ induced signalling cascades upon ARBE treatment, and the importance of IFN-γ in CD pathogenesis, our study further provides evidence that ARBE treatment might also be a promising approach for the treatment of CD patients.

## Supporting Information

S1 Fig 100 ng/ml is an optimal concentration for eliciting robust IFN-γ signalling pathway activation in THP-1 cells. We performed a concentration-action-curve stimulating THP-1 cells with 0 ng/ml, 1 ng/ml, 10 ng/ml, 100 ng/ml or 1000 ng/ml IFN-γ +/- 10 μg/ml ARBE. Detection of phospho-STAT1 is optimal with application of 100 ng/ml IFN-γ.
(TIFF)

S2 Fig Original uncropped and unadjusted Western blot from Fig 2. THP-1 cells were stimulated either with 10 μg/ml ARBE, IFN-γ and/or 0.5mM or 5mM Na_3_VO_4_ (PTP inhibitor). PTP inhibition did not abrogate ARBE-induced reduction of IFN-γ-mediated STAT1 phosphorylation. Ctr = control, IFN = IFN-γ, ACE = ARBE, I+A = IFN-γ + ARBE, p-STAT1 = phospho-STAT1, tSTAT1 = total STAT1.
(TIFF)

S3 Fig PTPN2 knockdown does not prevent ARBE-mediated reduction of IFN-γ-induced STAT1 phosphorylation. For PTPN2 knockdown, THP-1 cells were transfected with 100 pmol PTPN2 siRNA 36 h before stimulation. Pre-stimulation with10 μg/ml ARBE lasted 20 min and subsequent stimulation with 100 ng/ml IFN-γ (IFN) lasted 30 min. Untreated cells served as control group (Ctr). In THP-1 cells with PTPN2 knockdown co-stimulation with IFN+ARBE still provoked reduced STAT1 phosphorylation. Yet, these results were not significant.
(TIFF)

S4 Fig Overview of immunohistochemical staining of different molecular markers where detailed/enlarged images of Fig 3 are derived from. Representative pictures demonstrate colon biopsies before (week 1) and after (week 7) ARBE treatment either for patients reaching remission or not. **A** represents IFN-γ staining, **B** staining specific for IFGR2, **C** IFGR1 staining and **D** shows total STAT1 expression. A more detailed view is shown in Fig 3.
(TIFF)

S5 Fig IGFR2 expression in colon biopsy from control UC patient in remission is not suppressed. Pictures demonstrate representative sections of control colon biopsies derived from UC patients with active colitis, UC patients in remission under conventional therapy or from healthy persons. Low IFN-γ expression in healthy control colon tissue is detectable (**A**). IGFR2 expression is elevated in active colitis and colitis in remission control colon, whereas low expression is detectable in healthy controls (**B**). IGFR1 expression is hardly detectable in active colitis and colitis in remission controls, whereas IGFR1 expression is detectable in healthy control biopsies (**C**). For STAT1 expression low to moderate results were found in healthy control colon specimens (**D**).
(TIFF)

S6 Fig Serum cytokine concentrations during the study course differ depending on the remission status. Serum levels of different cytokines were measured each at week 1, week 3, week 5 and week 7 of the study course. Red dots represent results from patients that reached remission, black dots belong to patients that did not attain clinical remission. ARBE treatment did not affect the generally very low IFN-γ serum levels (n = 9) (**A**). TNF-α serum levels (only 5 participants featured serum concentrations above the detection level) decreased in patients reaching remission (n = 3; from 555 pg/ml down to 90 pg/ml), whereas they stayed stable when remission was not achieved (n = 2; from 3041.72 pg/ml to 2967 pg/ml) (**B**). IL-17A concentrations increased when remission was reached (n = 5; 0,53 pg/ml vs 0,8 pg/ml) or remained stable in patients without remission (n = 2) (**C**). IL-22 serum concentrations were stable during the ARBE treatment period not depending on the remission status (n = 11) (**D**). IL-10 concentrations were very low. Yet, a minimal increase was detectable in 5 of 8 patients (**E**). IL-13 measurement was inconclusive since concentrations above the detection threshold were present in only 3 patients (**F**).
(TIFF)

S7 Fig Overview of immunohistochemical staining of different molecular markers where detailed/enlarged images of Fig 4 are derived from. Representative pictures demonstrate colon biopsies before (week 1) and after (week 7) ARBE treatment either for patients reaching remission or not. Staining is specific for TNF-α (**A**), phospho-p65-NF-κB (**B**), IL-17A (**C**), IL-22 (**D**) and IL-10 (**E**), respectively. A more detailed view is shown in Fig 4.
(TIFF)

S8 Fig Elevated TNF-α expression in control biopsies from UC patients with active colitis. These representative pictures from control colon biopsies are derived from UC patients with active colitis or UC patients in remission under conventional therapy and healthy controls, respectively. As expected, TNF-α expression in UC patients with active colitis is elevated, whereas its expression is lowered when remission is attained. Healthy control colon depicts low TNF-α expression (**A**). phospho-p65-NF-κB expression is low in active colitis and colitis in remission control biopsies, while nearly no expression could be detected in healthy controls (**B**).
(TIFF)

S9 Fig Elevated IL-22 expression is found in control colitis. Pictures demonstrate representative sections of control colon biopsies derived from UC patients with active colitis, UC patients in remission under conventional therapy or from healthy controls. IL-17A expression in healthy colon is barely detectable (**A**). IL-22 expression levels are elevated in active colitis control as well as in colitis in remission under conventional therapy biopsies, whereas healthy controls feature lower IL-22 expression (**B**). IL-10 expression in healthy controls is readily detectable (**C**).
(TIFF)

S1 File Analysis of the ARBE powder used in these experiments. (PDF)

S2 File Raw data underlying cellular and ELISA experiments. (XLSX)

S1 Table Quantification of immunohistochemistry results performed by blinded scoring. (XLSX)
