# Supplementary figures and images for: Bilberry-Derived Anthocyanins Modulate Cytokine Expression in the Intestine of Patients with Ulcerative Colitis
Source: PLoS One. 2016 May 6;11(5):e0154817. doi: 10.1371/journal.pone.0154817 (PMC4859486; doi:10.1371/journal.pone.0154817)

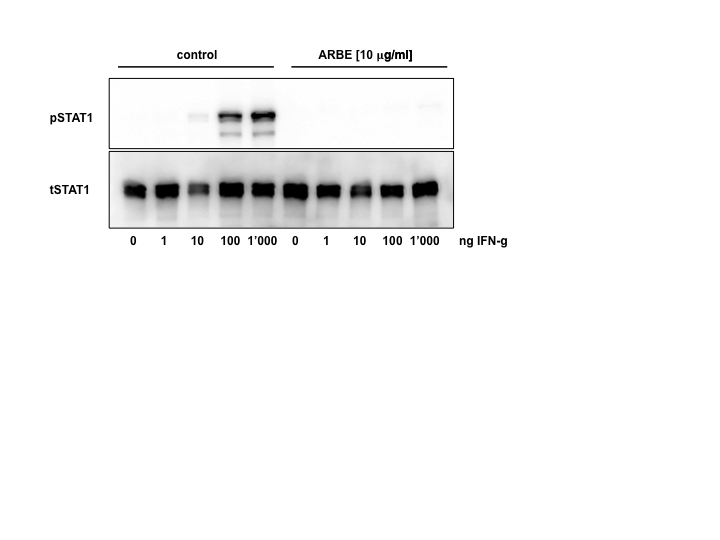

Supplement: S1 Fig — We performed a concentration-action-curve stimulating THP-1 cells with 0 ng/ml, 1 ng/ml, 10 ng/ml, 100 ng/ml or 1000 ng/ml IFN-γ +/- 10 μg/ml ARBE. Detection of phospho-STAT1 is optimal with application of 100 ng/ml IFN-γ. (TIFF) [file pone.0154817.s001.tiff]

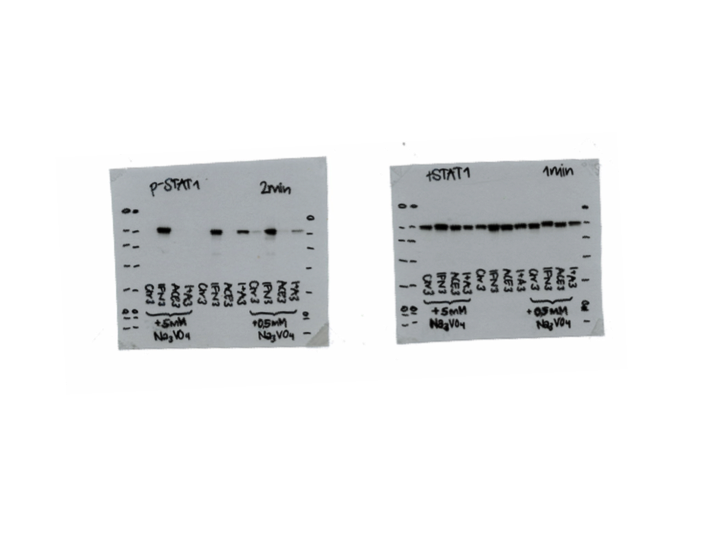

Supplement: S2 Fig — THP-1 cells were stimulated either with 10 μg/ml ARBE, IFN-γ and/or 0.5mM or 5mM Na3VO4 (PTP inhibitor). PTP inhibition did not abrogate ARBE-induced reduction of IFN-γ-mediated STAT1 phosphorylation. Ctr = control, IFN = IFN-γ, ACE = ARBE, I+A = IFN-γ + ARBE, p-STAT1 = phospho-STAT1, tSTAT1 = total STAT1. (TIFF) [file pone.0154817.s002.tiff]

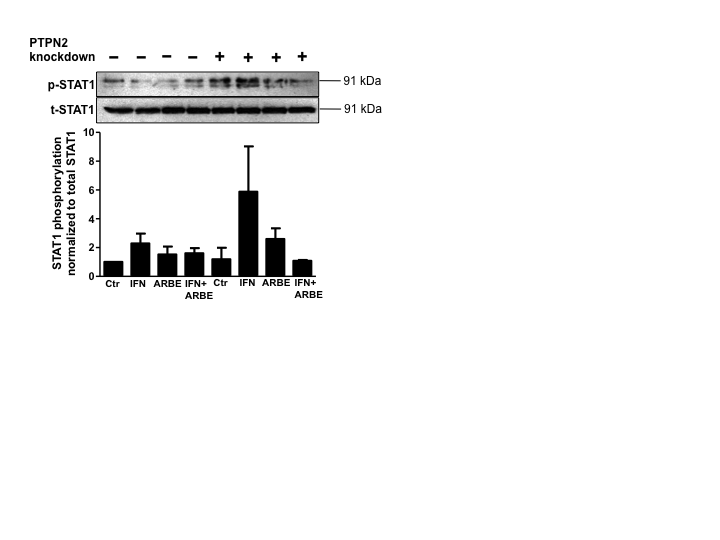

Supplement: S3 Fig — For PTPN2 knockdown, THP-1 cells were transfected with 100 pmol PTPN2 siRNA 36 h before stimulation. Pre-stimulation with10 μg/ml ARBE lasted 20 min and subsequent stimulation with 100 ng/ml IFN-γ (IFN) lasted 30 min. Untreated cells served as control group (Ctr). In THP-1 cells with PTPN2 knockdown co-stimulation with IFN+ARBE still provoked reduced STAT1 phosphorylation. Yet, these results were not significant. (TIFF) [file pone.0154817.s003.tiff]

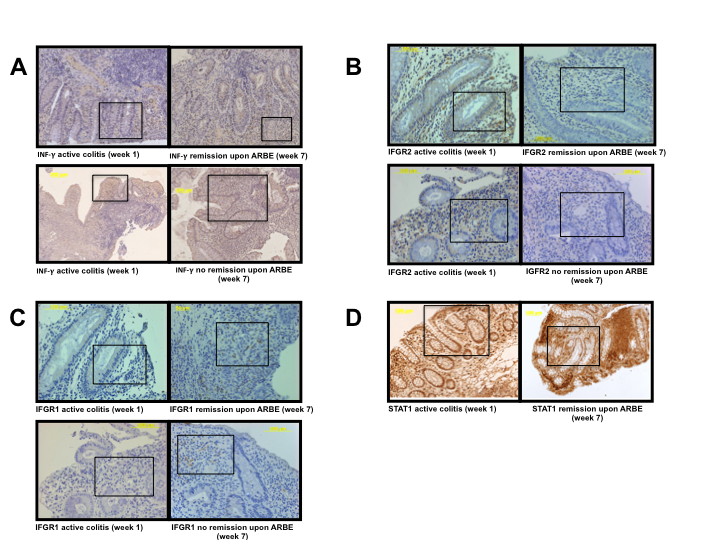

Supplement: S4 Fig — Representative pictures demonstrate colon biopsies before (week 1) and after (week 7) ARBE treatment either for patients reaching remission or not. A represents IFN-γ staining, B staining specific for IFGR2, C IFGR1 staining and D shows total STAT1 expression. A more detailed view is shown in Fig 3. (TIFF) [file pone.0154817.s004.tiff]

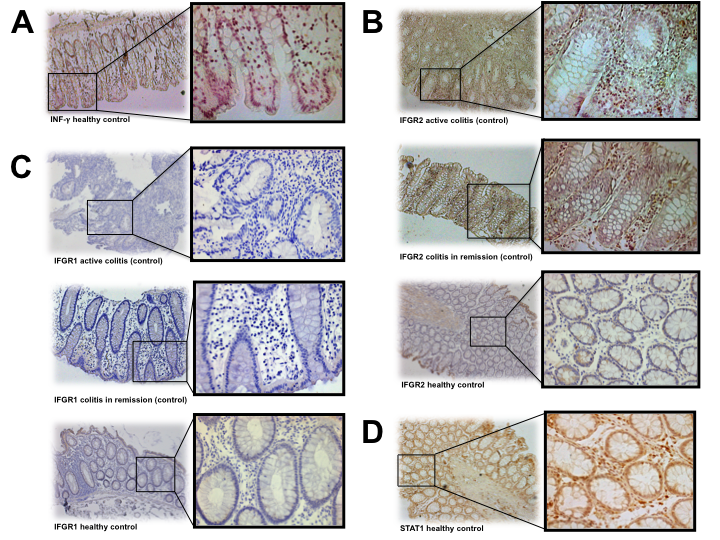

Supplement: S5 Fig — Pictures demonstrate representative sections of control colon biopsies derived from UC patients with active colitis, UC patients in remission under conventional therapy or from healthy persons. Low IFN-γ expression in healthy control colon tissue is detectable (A). IGFR2 expression is elevated in active colitis and colitis in remission control colon, whereas low expression is detectable in healthy controls (B). IGFR1 expression is hardly detectable in active colitis and colitis in remission controls, whereas IGFR1 expression is detectable in healthy control biopsies (C). For STAT1 expression low to moderate results were found in healthy control colon specimens (D). (TIFF) [file pone.0154817.s005.tiff]

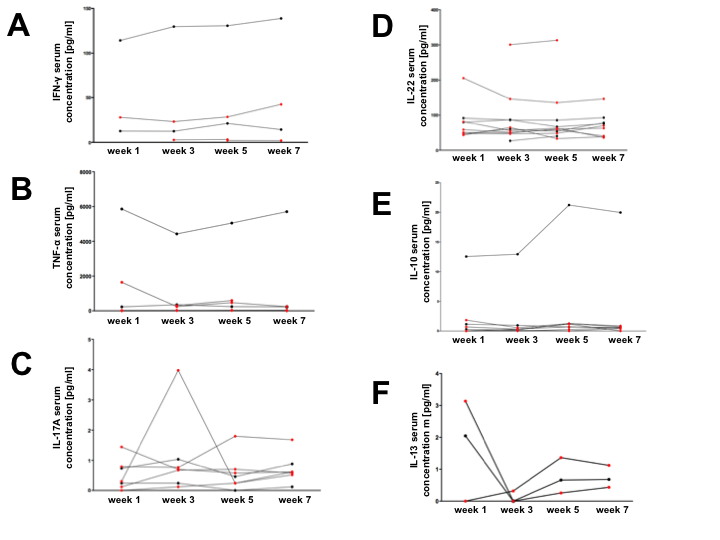

Supplement: S6 Fig — Serum levels of different cytokines were measured each at week 1, week 3, week 5 and week 7 of the study course. Red dots represent results from patients that reached remission, black dots belong to patients that did not attain clinical remission. ARBE treatment did not affect the generally very low IFN-γ serum levels (n = 9) (A). TNF-α serum levels (only 5 participants featured serum concentrations above the detection level) decreased in patients reaching remission (n = 3; from 555 pg/ml down to 90 pg/ml), whereas they stayed stable when remission was not achieved (n = 2; from 3041.72 pg/ml to 2967 pg/ml) (B). IL-17A concentrations increased when remission was reached (n = 5; 0,53 pg/ml vs 0,8 pg/ml) or remained stable in patients without remission (n = 2) (C). IL-22 serum concentrations were stable during the ARBE treatment period not depending on the remission status (n = 11) (D). IL-10 concentrations were very low. Yet, a minimal increase was detectable in 5 of 8 patients (E). IL-13 measurement was inconclusive since concentrations above the detection threshold were present in only 3 patients (F). (TIFF) [file pone.0154817.s006.tiff]

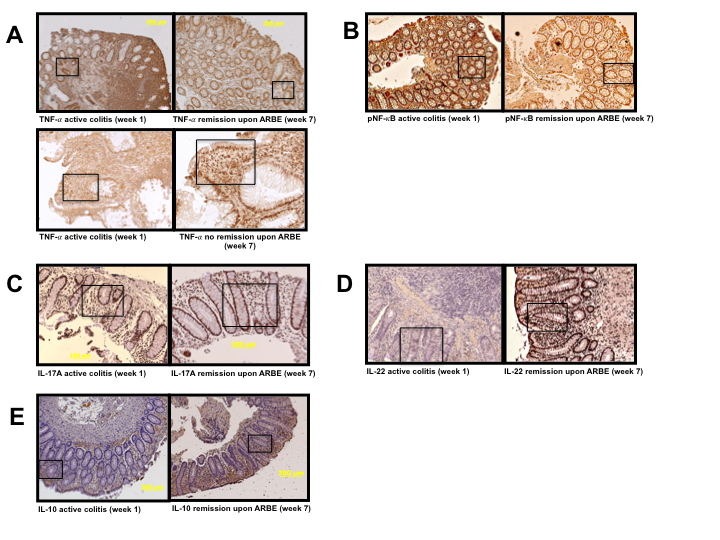

Supplement: S7 Fig — Representative pictures demonstrate colon biopsies before (week 1) and after (week 7) ARBE treatment either for patients reaching remission or not. Staining is specific for TNF-α (A), phospho-p65-NF-κB (B), IL-17A (C), IL-22 (D) and IL-10 (E), respectively. A more detailed view is shown in Fig 4. (TIFF) [file pone.0154817.s007.tiff]

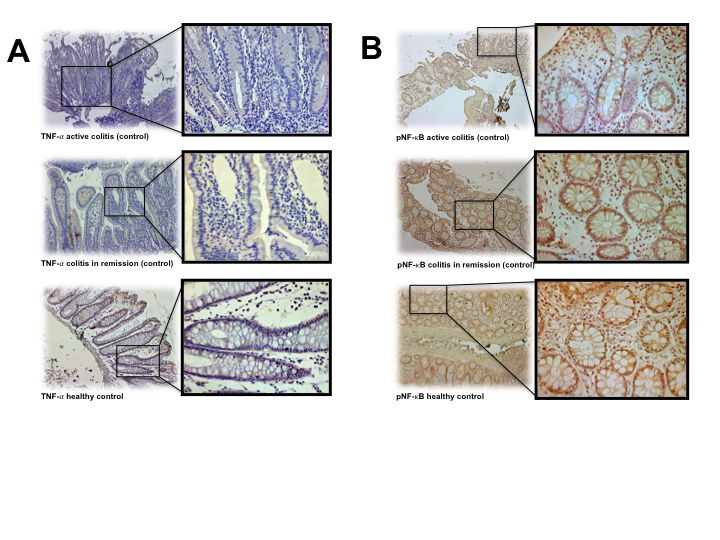

Supplement: S8 Fig — These representative pictures from control colon biopsies are derived from UC patients with active colitis or UC patients in remission under conventional therapy and healthy controls, respectively. As expected, TNF-α expression in UC patients with active colitis is elevated, whereas its expression is lowered when remission is attained. Healthy control colon depicts low TNF-α expression (A). phospho-p65-NF-κB expression is low in active colitis and colitis in remission control biopsies, while nearly no expression could be detected in healthy controls (B). (TIFF) [file pone.0154817.s008.tiff]

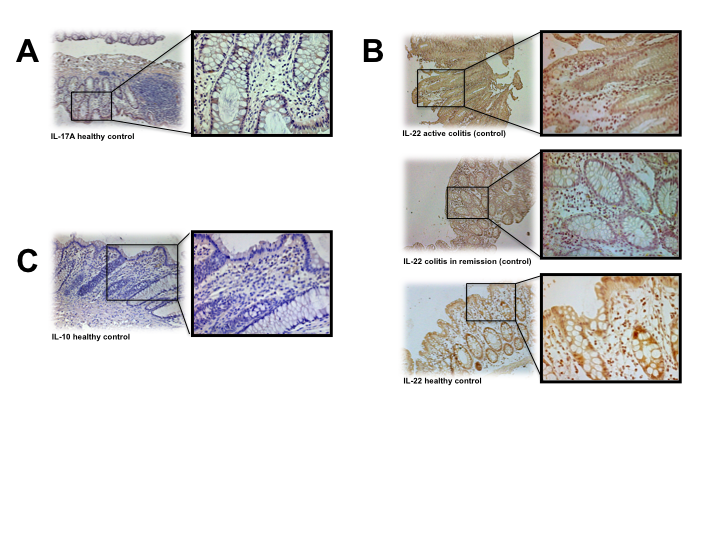

Supplement: S9 Fig — Pictures demonstrate representative sections of control colon biopsies derived from UC patients with active colitis, UC patients in remission under conventional therapy or from healthy controls. IL-17A expression in healthy colon is barely detectable (A). IL-22 expression levels are elevated in active colitis control as well as in colitis in remission under conventional therapy biopsies, whereas healthy controls feature lower IL-22 expression (B). IL-10 expression in healthy controls is readily detectable (C). (TIFF) [file pone.0154817.s009.tiff]
