# Supplementary material for: Bilberry-Derived Anthocyanins Modulate Cytokine Expression in the Intestine of Patients with Ulcerative Colitis
Source: PLoS One. 2016 May 6;11(5):e0154817. doi: 10.1371/journal.pone.0154817 (PMC4859486; doi:10.1371/journal.pone.0154817)
Supplement: S1 File — (PDF) [file pone.0154817.s010.pdf]

symrise
always inspiring more...

Formulation: **9/0R2575/00**
Category : **Confectionary**
Type : **Bilberry Fruit Concentrate With Dried Billberries**

Position	Raw Material	%
**1**	**(602589) BLUEBERRY DRIED SIEVED**	**59,630**
**2**	**(122112) BLUEBERRY JUICE CONC. 65BX**	**25,900**
3	RASINS	4,650
4	SUGAR BEET SYRUP	4,650
5	ICING SUGAR	4,650
6	ACESULFAM K	0,020
**7**	**(653517) Bilberry/Blueberry Flavouring**	**0,500**
		100,000

Position	Suppliers
1,2,7	Symrise GmbH & Co. KG, DE-37603 Holzminden

Production Method

Preparation

Per one daily dosage 4 portions, each 40g, are recommended, (corresponding 95 g dried billberries).

Our suggestions have been carefully tested, but are without obligation. It is the duty of the manufacturer to test the finished product and check the patent situation. The list of suppliers is not a value of judgment.

BILBERRY FRUIT PREPARATION
QUANTITATION OF ANTHOCYANINS

symrise
always inspiring more...

CONDITIONS

- Gradient elution: ethanol/water/formic acid
- Column material: fused core
- Extraction solution: ethanol/water/phosphoric acid/hydrochloric acid

HPLC OF FRUIT CONCENTRATE

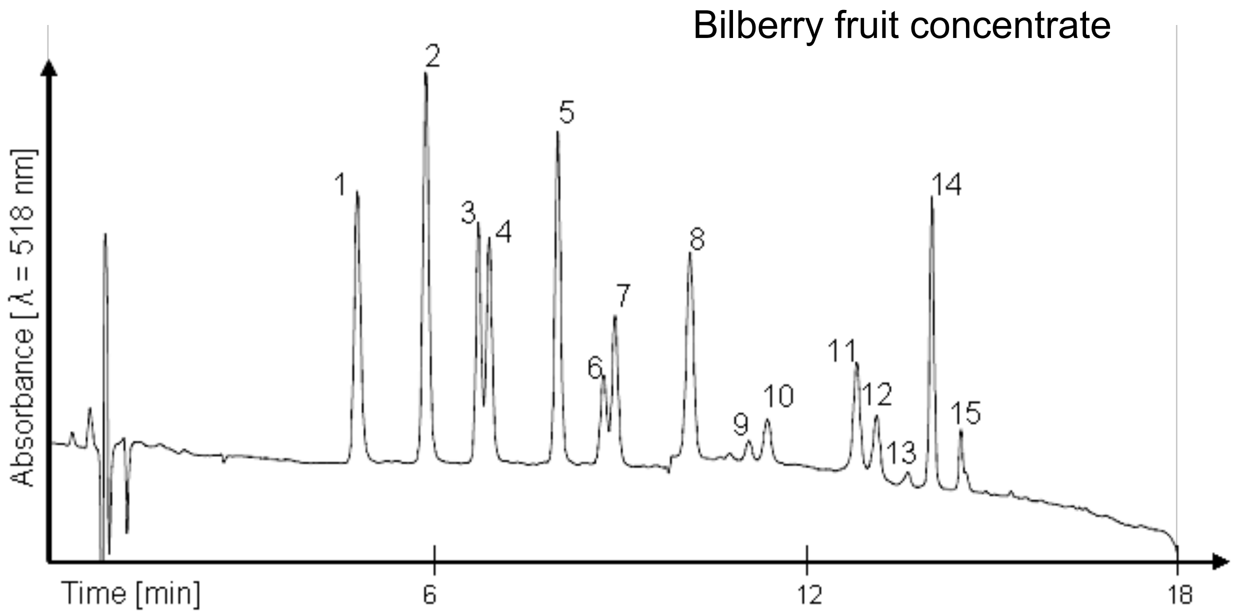

Peak	Anthocyanin
1	Del-3-Gal
2	Del-3-Glu
3	Cy-3-Gal
4	Del-3-Ara
5	Cy-3-Glu
6	Pet-3-Gal
7	Cy-3-Ara
8	Pet-3-Glu
9	Peo-3-Gal
10	Pet-3-Ara
11	Peo-3-Glu
12	Mal-3-Gal
13	Peo-3-Ara
14	Mal-3-Glu
15	Mal-3-Ara

The anthocyanin pattern of the Bilberry fruit preparation is comparable to the Actiplants® Bilberry extract 25 %
